# Regional sex differences in neurochemical profiles of healthy mice measured by magnetic resonance spectroscopy at 9.4 tesla

**DOI:** 10.3389/fnins.2023.1278828

**Published:** 2023-10-25

**Authors:** Ivan Tkáč, Tiankai Xie, Nitya Shah, Sarah Larson, Janet M. Dubinsky, Rocio Gomez-Pastor, Hayley S. McLoughlin, Harry T. Orr, Lynn E. Eberly, Gülin Öz

**Affiliations:** ^1^Center for Magnetic Resonance Research, Department of Radiology, University of Minnesota, Minneapolis, MN, United States; ^2^Division of Biostatistics, School of Public Health, University of Minnesota, Minneapolis, MN, United States; ^3^Department of Neuroscience, University of Minnesota, Minneapolis, MN, United States; ^4^Department of Neurology, University of Michigan, Ann Arbor, MI, United States; ^5^Department of Laboratory Medicine and Pathology, University of Minnesota, Minneapolis, MN, United States

**Keywords:** metabolite, taurine, glucose, total creatine, ascorbate, glutamate

## Abstract

**Objective:**

To determine sex differences in the neurochemical concentrations measured by *in vivo* proton magnetic resonance spectroscopy (^1^H MRS) of healthy mice on a genetic background commonly used for neurodegenerative disease models.

**Methods:**

^1^H MRS data collected from wild type mice with C57BL/6 or related genetic backgrounds in seven prior studies were used in this retrospective analysis. To be included, data had to be collected at 9.4 tesla magnetic field using advanced ^1^H MRS protocols, with isoflurane anesthesia and similar animal handling protocols, and a similar number of datasets from male and female mice had to be available for the brain regions analyzed. Overall, 155 spectra from female mice and 166 spectra from male mice (321 in total), collected from six brain regions (brainstem, cerebellum, cortex, hippocampus, hypothalamus, and striatum) at various ages were included.

**Results:**

Concentrations of taurine, total creatine (creatine + phosphocreatine), ascorbate, glucose and glutamate were consistently higher in male vs. female mice in most brain regions. Striatum was an exception with similar total creatine in male and female mice. The sex difference pattern in the hypothalamus was notably different from other regions. Interaction between sex and age was significant for total creatine and taurine in the cerebellum and hippocampus.

**Conclusion:**

Sex differences in regional neurochemical levels are small but significant and age-dependent, with consistent male–female differences across most brain regions. The neuroendocrine region hypothalamus displays a different pattern of sex differences in neurochemical levels. Differences in energy metabolism and cellular density may underlie the differences, with higher metabolic rates in females and higher osmoregulatory and antioxidant capacity in males.

## 1. Introduction

Mouse models of human neurodegenerative diseases have been essential in understanding disease mechanisms and evaluating promising therapeutics in preclinical trials (Marin-Moreno et al., 2023). Noninvasive methods that can be utilized to characterize mouse models and that can also be translated to human subjects are particularly valuable in bridging mouse and human findings and expediting the translational pipeline.

*In vivo* proton magnetic resonance spectroscopy (^1^H MRS) is one such method that has been widely used for non-invasive monitoring of neurochemistry in mouse models (Choi et al., 2007; Tkáč, 2016; Lanz et al., 2021). ^1^H MRS provides non-invasive access to the endogenous concentrations of up to 20 neurochemicals, including metabolites primarily localized in neurons or glia, neurotransmitters, antioxidants and indicators of energy and membrane lipid metabolism (Duarte et al., 2012). Judicious translation of MRS findings from mouse models to humans necessitates consideration of sex as a biological variable. While the research landscape has been changing since the Sex as a Biological Variable policy of the National Institutes of Health went into effect in January 2016, many prior preclinical studies excluded female animals to eliminate potential variability in experimental outcomes associated with the estrous cycle and to obviate the need to control for the phase of estrous cycle in experimental design (Simpson and Kelly, 2012).

Sex differences in neurochemistry, e.g., in neurotransmitter systems such as dopamine and serotonin, have been documented using invasive methods in animal models and postmortem human brain (Cosgrove et al., 2007). However, detailed understanding of sex differences in the levels of the high concentration (millimolar) neurochemicals that are detectable by *in vivo* ^1^H MRS is lacking. Existing MRS literature in humans reported conflicting findings, with some studies reporting no neurochemical differences between men and women (Pouwels and Frahm, 1998; Nagae-Poetscher et al., 2004), while others indicating regional differences in select metabolites such as GABA, glutamate, choline containing compounds and total creatine (Sanacora et al., 2003; Hadel et al., 2013; Endres et al., 2016). As for rodent ^1^H MRS literature, a recent report outlined multiple neurochemical differences between male and female Fisher 344 rats (Fowler et al., 2021). For mouse models however, there is only one report of sex differences in neurochemical levels (Duarte et al., 2014). The primary focus of that study was age-related changes in the neurochemical profile of mice on the C57BL/6 background, the most widely used strain in biomedical research (Bryant, 2011). In a secondary analysis, the authors reported higher taurine levels in the cortex, hippocampus, and striatum of male relative to age-matched female mice, and indicated differences in additional metabolites, but without providing the direction of the differences.

A detailed understanding of sex differences in ^1^H MRS-detectable neurochemical levels is important for design of future preclinical studies and valid translation of findings from neurodegenerative disease models to humans. Furthermore, the lifetime risk for cognitive decline and Alzheimer disease is higher in women than men (Ferretti et al., 2018; Alzheimer’s Association, 2021). Therefore, our goal in this study was to determine sex differences in six brain regions relevant to multiple neurodegenerative diseases, namely the brainstem, cerebellum, cortex, hippocampus, hypothalamus, and striatum, in mice with genetic backgrounds commonly used for neurodegenerative disease models.

## 2. Methods

### 2.1. Study design

To determine sex differences in regional neurochemical profiles in the mouse brain, we used ^1^H MRS data from our previous studies of mouse models of neurodegenerative and metabolic diseases, including spinocerebellar ataxias (SCA1 and SCA3), Huntington disease (HD) and mucopolysaccharidosis (MPS1) (Zacharoff et al., 2012; Tkáč et al., 2016; Friedrich et al., 2018; McLoughlin et al., 2023; Zarate et al., 2023). In addition, ^1^H MRS data were used from two other studies investigating the effects of maternal obesity on offspring (Kulhanek et al., 2022) and of chronic exposure to ultra-high magnetic field (Tkáč et al., 2021; Table 1). Sex differences in hippocampal neurochemical levels observed in mice scanned in the ultra-high field exposure study (Tkáč et al., 2021) (40 mice scanned at 2 months and 40 others scanned at 3 months of age) were presented at a conference (Larson and Tkáč, 2018) but have not been published previously. All mice had C57BL/6 or related genetic backgrounds. We only included MRS data acquired from wild type (WT) mice that did not undergo any treatment in this retrospective analysis. These studies provided ^1^H MRS data from six brain regions (brainstem, cerebellum, cortex, hippocampus, hypothalamus, and striatum) at various ages. Data from a similar number of male and female mice were required for each brain region to be included. Overall, 155 MR spectra from female mice and 166 MR spectra from male mice (321 in total) were included in the analysis (Table 1).

**Table 1:** Data that were used to analyze sex differences in wild type mice.

Brain region	Background	Age (weeks)	Sample Size		MRS method	Citation
			N (Female)	N (Male)		
Hippocampus	C57BL/6	8	20	20	STEAM	Tkáč et al. (2021)
		12	20	20		
Hippocampus	C57BL/6	34	6	6	STEAM	Tkáč et al. (2016)
Cerebellum		34	6	6		
Cerebellum	C57BL/6	18	4	7	LASER	Friedrich et al. (2018)
		28	6	9		
Brainstem		18	4	7		
		28	6	9		
Cerebellum	C57BL/6 J	16	9	9	LASER	McLoughlin et al. (2023)
		34	5	6		
		40	2	3		
Brainstem		16	8	9		
		34	5	6		
		40	2	3		
Striatum	129/SvEv-C57BL/6 J	13	8	5	LASER	Zarate et al. (2023)
		26	8	1		
		53	3	2		
Striatum	C57BL6/CBA	4	4	4	STEAM	Zacharoff et al. (2012)
		8	4	4		
		12	4	4		
		15	2	4		
Cortex		4	4	4		
		8	4	4		
		12	4	4		
		15	2	4		
Hypothalamus	C57BL/6 J	16	5	6	LASER	Kulhanek et al. (2022)
TOTAL number of spectra			155	166		

### 2.2. MR methods

^1^H MRS data of animal models included in this study were collected over 15 years. Despite this long period, the protocols, including animal handling, MRS data acquisition and processing, were sufficiently consistent to allow the combined analysis. The main difference between the studies included in this project was the ^1^H MRS localization sequence, which was either STEAM (Tkáč et al., 1999) or LASER (Garwood and DelaBarre, 2001), and the associated acquisition parameters.

#### 2.2.1. Data acquisition

*In vivo* ^1^H MR spectra were collected from spontaneously breathing mice under inhalational anesthesia (isoflurane, ~3% for induction and 1.4–2.0% for maintenance in a 50:50 mixture of N_2_O and O_2_). Body temperature was maintained at 36 – 37°C using circulating warm water surrounding the animal holder and in some studies by an additional heating fan controlled by feedback from a fiber-optic rectal thermometer. The respiration rate was continuously monitored (SA Instruments, Stony Brook, NY) and maintained at 70–100 per minute by adjusting the isoflurane level. The total scanning time of each animal did not exceed 90 min.

**Figure 1:**
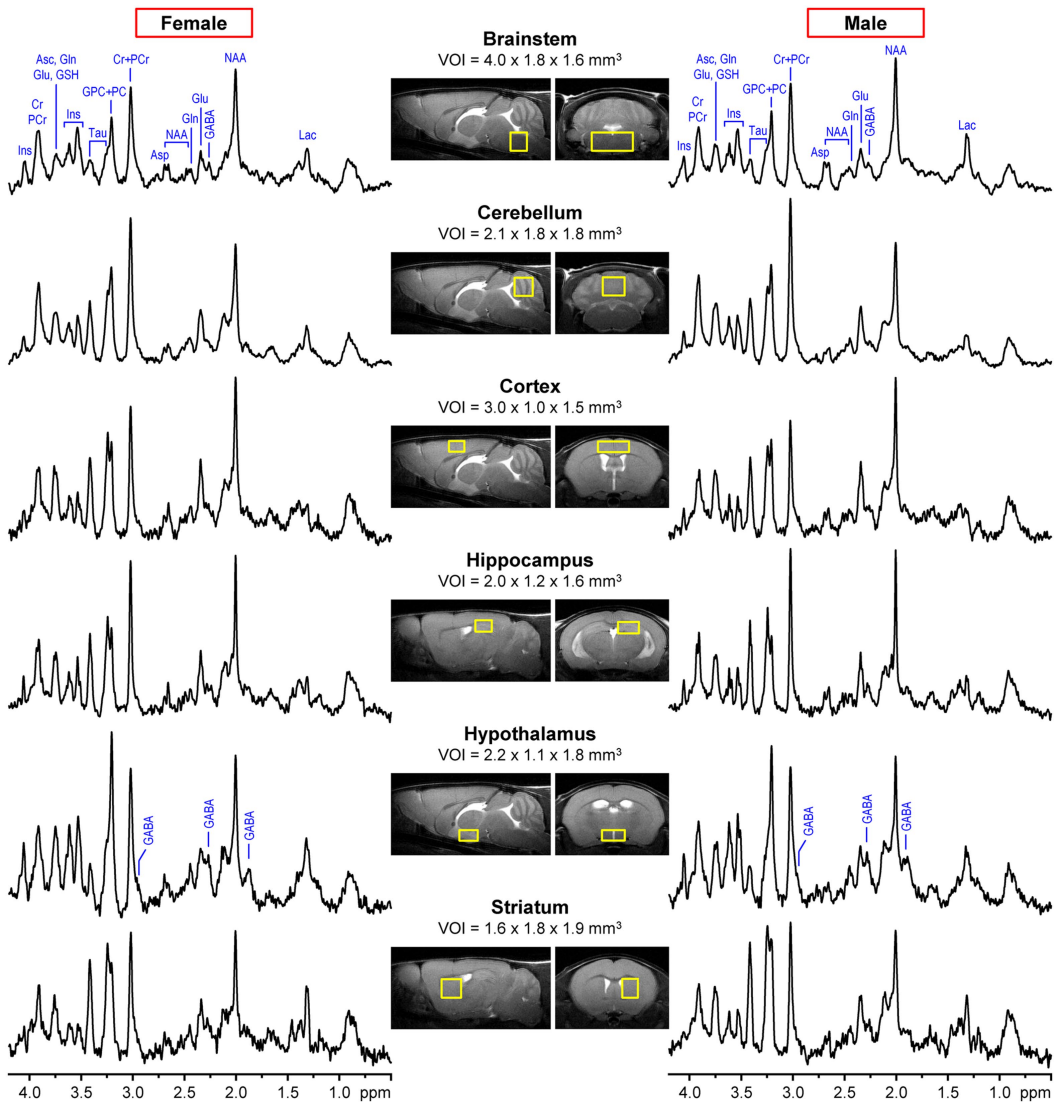
Examples of representative ^1^H MR spectra of male and female mice acquired from six different brain regions at 9.4T using LASER (TE = 15 ms, brainstem and cerebellum) or STEAM (TE = 2 ms, cortex, hippocampus, hypothalamus, striatum). Fast *spin-echo* (FSE) images in axial and sagittal orientations show the typical size and location of the volumes of interest (VOI) in each brain region. Metabolites with distinct peaks are labeled in the brainstem spectra. In addition, all three resonances of GABA are labeled in the hypothalamus spectra to highlight the high concentration of this metabolite in this brain region.

All studies were performed using a 9.4 T/31 cm horizontal bore magnet (Varian/Magnex Scientific, Yarnton, UK) equipped with a 15-cm gradient/shim coil (Resonance Research, Billerica, MA, USA) and interfaced originally to INOVA and later to a DirectDrive console (Agilent/Varian, Palo Alto, CA, USA). All MR data were acquired using quadrature transmit/receive surface radiofrequency (RF) coils with two geometrically decoupled single-turn coils of either 10 or 14 mm diameter. The homogeneity of the static magnetic field B_0_ was adjusted by FASTMAP automatic shimming (Gruetter and Tkáč, 2000). The ^1^H MR spectra were acquired using either an ultra-short echo-time STEAM (Tkáč et al., 1999) (echo time (TE) = 2 ms, repetition time (TR) = 5 s, number of averages = 160–320) or LASER (Garwood and DelaBarre, 2001) (TE =15 ms, TR = 5 s, number of averages = 160–240) localization sequences. Water signal was suppressed using the VAPOR technique (Tkáč et al., 1999). ^1^H MR spectra with unsuppressed water signal (number of averages = 4–8) were used for eddy current correction and as a reference for metabolite quantification. Multislice fast spin-echo (FSE, echo train length = 8, echo spacing = 12 ms) MRI in axial and sagittal orientations was used for positioning of the volume-of-interest (VOI) in targeted brain regions. Typical locations and sizes of VOIs in the six brain regions are shown in Figure 1.

#### 2.2.2. Data analysis

^1^H MR spectra acquired and saved as single shots or averages of 8 scans were first corrected for frequency and phase variations, then summed and finally corrected for residual eddy currents using the unsuppressed water signal (Klose, 1990). Brain metabolites were quantified from averaged ^1^H MR spectra using LCModel (Provencher, 2001) with simulated basis sets (for STEAM or LASER) that also included experimentally measured spectra of fast relaxing macromolecules. The unsuppressed water signal was used as an internal reference, assuming 80–82% brain water content. Out of 20 metabolites included in the basis sets, only metabolites that were consistently quantified with the Cramér-Rao lower bounds (CRLB) below 50% or with mean CRLB ≤20% were included for further analysis. Namely, we adhered to CRLB reporting criteria that were used in the papers from which data were gathered for consistency in the reported metabolites with prior literature. Metabolites reported in this retrospective analysis include alanine (Ala), aspartate (Asp), ascorbate (Asc), γ-aminobutyric acid (GABA), glucose (Glc), glutamine (Gln), glutamate (Glu), glutathione (GSH), glycine (Gly), *myo*-inositol (myo-Ins), lactate (Lac), *N*-acetylaspartate (NAA), *N*-acetylaspartylglutamate (NAAG), phosphoethanolamine (PE), taurine (Tau), and the sums of glycerophosphocholine and phosphocholine (GPC + PC) and creatine and phosphocreatine (Cr + PCr).

### 2.3. Statistical analysis

To analyze differences in metabolite concentrations between male and female mice, existing data from seven previous studies were used and contained information from six brain regions (brainstem, cerebellum, cortex, hippocampus, hypothalamus, and striatum) that were acquired using two different MRS localization techniques (LASER and STEAM). Analyses were done separately for each brain region, combining data across available studies within each brain region and keeping those that met the CRLB reporting criteria described above. To avoid the assumption that metabolites changed linearly with age, age (in weeks) was treated as a categorical predictor rather than a continuous variable. Mice were scanned at one or more of 12 ages, ranging from 4 to 53 weeks, in the included studies and each age available for a brain region represented one category.

Differences between male and female metabolite concentrations (male minus female) were analyzed separately by brain region. Because all measurements in the hypothalamus were taken at the age of 16 weeks and using a single MRS method (LASER), the differences in metabolite concentrations between males and females were compared using t-tests. The concentrations of metabolites in the hippocampus were modeled using multiple linear regression (MLR), using sex as a predictor and adjusting for the effect of age.

MLR models were also fit for the remaining regions. For metabolites in the brainstem and the cortex, concentrations were modeled using sex as predictor and adjusting for age. For the cerebellum and the striatum, we also included an adjustment for the MRS method (STEAM, LASER). While these four regions had data collected at multiple ages for the same mice, longitudinal linear mixed-effects models with a random effect for mouse were attempted but did not successfully converge because the mouse variance component was too close to zero.

To address the multiplicity issue arising from analyzing many metabolites in each region, the Holm–Bonferroni method (Holm, 1979) was implemented to sequentially adjust the value of ps and control each region-specific family-wise error rate.

## 3. Results

### 3.1. Characterization of ^1^H MR spectra used in sex difference analysis

Figure 1 shows the typical size and location of VOIs in six different mouse brain regions selected for ^1^H MRS data acquisition. This figure also demonstrates the spectral quality that was consistently achieved in male and female mice across 7 independent MRS studies (Table 1). Only MRS data from untreated WT animals in these studies were included in this retrospective analysis. The consistency in spectral quality between MR spectra acquired from male and female mice is documented by estimated metabolite linewidths provided by the standard output of the LCModel analysis (Supplementary Table S1). As expected, average metabolite linewidths were different between brain regions due to intrinsic tissue properties, namely microscopic B_0_ magnetic field inhomogeneities (Juchem et al., 2021), with hippocampus and the striatum displaying the narrowest and brainstem the broadest linewidths. Linewidths were not significantly different between MRS data from male and female mice for any brain region.

Moreover, unique spectral patterns characteristic of each brain region are apparent in Figure 1, e.g., high Cr + PCr in the cerebellum, high Tau in the cortex, hippocampus and striatum, low Tau and high GPC + PC and GABA in the hypothalamus. However, small differences between spectra from male and female mice are not easily discernable.

### 3.2. Regional sex differences in neurochemical profiles

**Figure 2:**
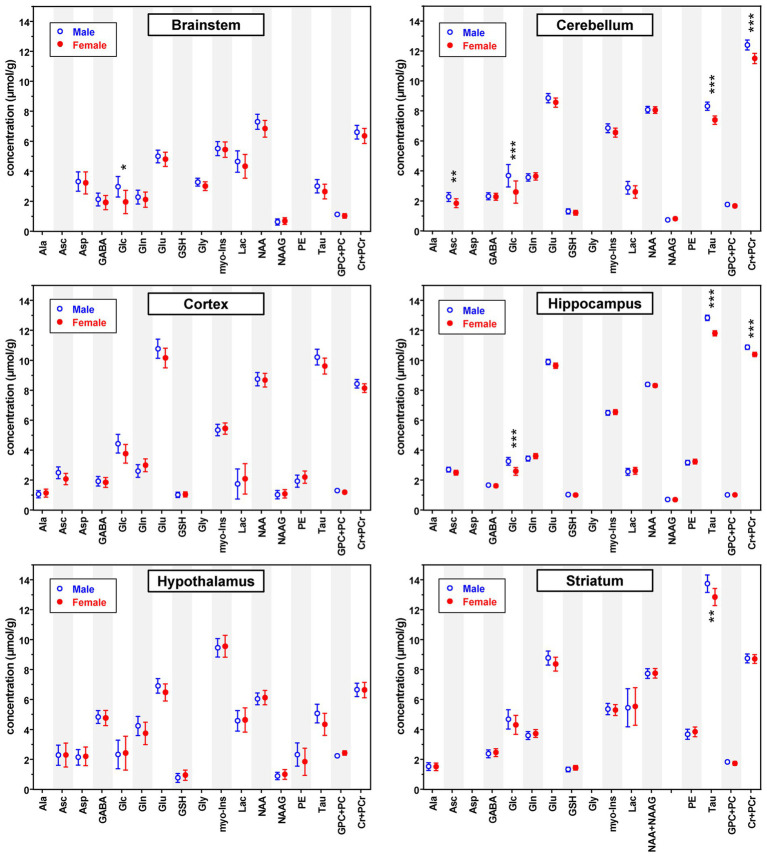
Predicted neurochemical mean concentrations (μmol/g) and 95% confidence intervals for healthy male and female mice in each brain region at an example age (brainstem: 18 weeks; cerebellum: 16 weeks; cortex: 12 weeks; hippocampus: 12 weeks; hypothalamus: 16 weeks; striatum: 12 weeks) across methods. Concentrations predicted by the statistical models (hippocampus, cortex and brainstem values adjusted for categorical age; striatum and cerebellum values adjusted for MRS method and categorical age; hypothalamus values unadjusted) are shown. Different example ages are shown for different regions because age was treated as a categorical predictor and the ages used for prediction were constrained to the existing ages within the statistical model. The value of ps shown are Holm-Bonferroni corrected for multiple testing across metabolites within region. *p < 0.05; **p < 0.005; ***p < 0.0005.

**Figure 3:**
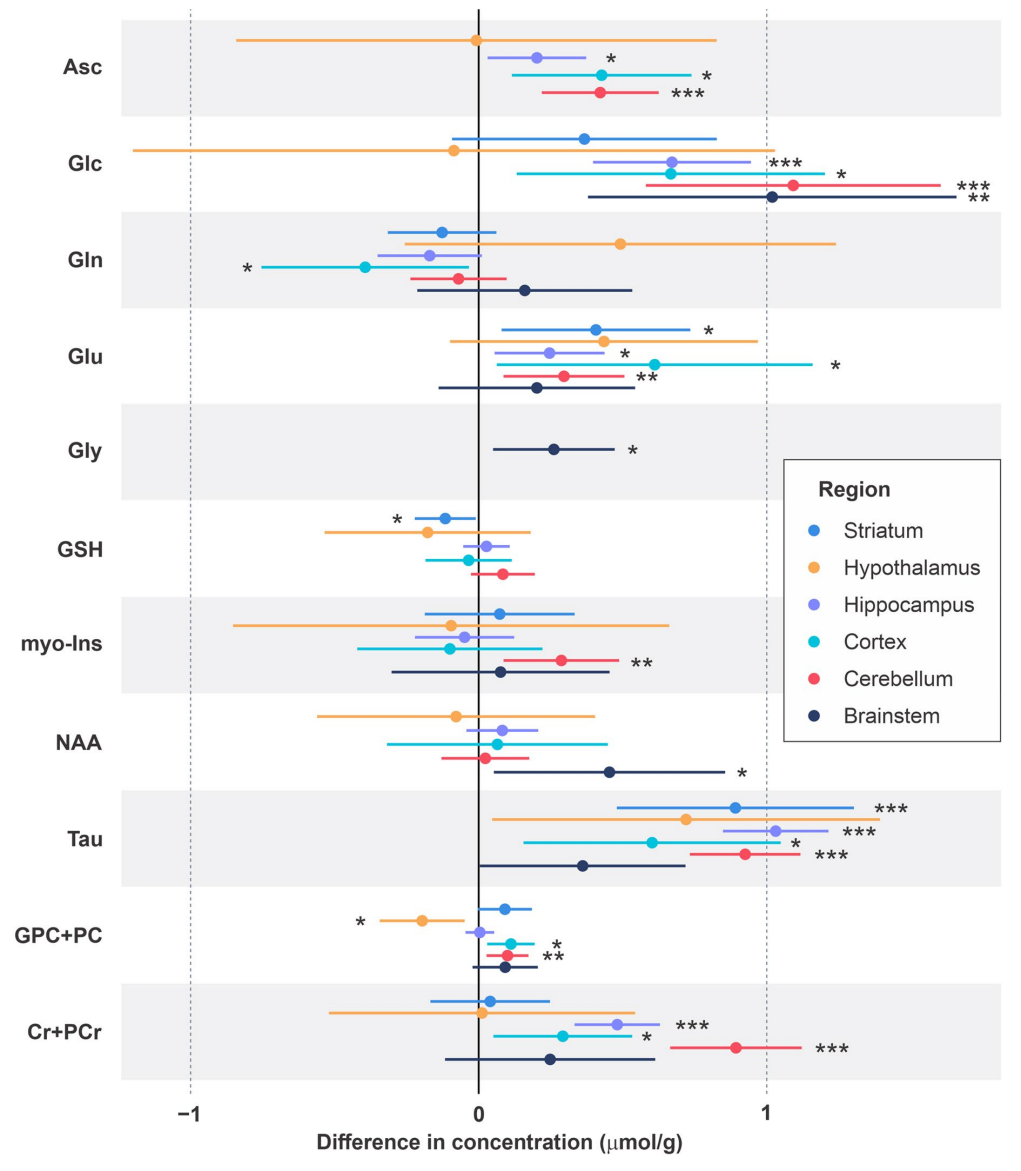
Differences in neurochemical concentrations for male vs. female mice across ages and methods. The neurochemicals that were significantly different in at least one brain region are depicted. Mean differences (male minus female concentrations) are shown along with 95% confidence intervals and uncorrected value of *p*s. *p < 0.05; **p < 0.005; ***p < 0.0005.

Quantitative analysis of the MR spectra revealed differences between neurochemical profiles of male vs. female mice (Figure 2). Consistent with the spectral pattern characteristic for each brain region, neurochemical profiles were unique to each brain region and different from each other. On the other hand, the differences in metabolite levels between male and female mice were relatively small, but highly significant, especially in brain regions with large sample size, namely the hippocampus and cerebellum (Figure 3; Supplementary Table S2).

Figure 3 depicts mean differences in the concentrations of metabolites that were significantly different between male and female mice in at least one brain region. Based on Holm-Bonferroni adjusted value of ps, Asc concentration was higher in males than females in the cerebellum (*p* < 0.002), Glc was higher in males in the brainstem (*p* < 0.05), cerebellum (*p* < 0.001) and hippocampus (*p* < 0.0001), Tau was higher in males in the cerebellum (*p* < 10^−11^), hippocampus (p < 10^−16^) and striatum (p < 0.002), and Cr + PCr was higher in males in the cerebellum (p < 10^−8^) and hippocampus (p < 10^−6^). While these differences in metabolite concentrations between male and female mice are brain region specific, sex differences in metabolites showed very consistent trends across regions even when not statistically significant after Holm correction. Namely, higher levels of Asc, Glc, Tau and total creatine (Cr + PCr) in male vs. female mice were apparent nearly in all brain regions. Striatum was an exception with similar Cr + PCr in male and female mice. In addition, glutamate levels were higher in males across regions. The sex difference pattern in the hypothalamus was notably different than other regions, with only Tau and Glu showing the same trend as in other regions and lower GPC + PC in male vs. female mice. Finally, Gly was detected reliably only in the brainstem due to its high concentration in this brain region and tended to be higher in male mice.

We further analyzed age-sex interactions in brain regions with data available at multiple ages (Table 1). The MLR analyses revealed statistically significant interactions between sex and age for several metabolites (Table 2). Notably, the age-sex interactions were highly significant for total creatine and Tau in the cerebellum and hippocampus. The sex differences (mostly higher concentrations in males) were consistent across ages and the age-sex interaction was manifested by differences that became larger or smaller with age.

**Table 2:** Results of the multiple linear regression (MLR) analysis of MRS data to define age related metabolite sex differences.^a^

Brain region	Metabolite	Sex coeff.^b^	Lower bound	Upper bound	*p*-value	*p*-value adjusted	Statistical model^c^
Brainstem	Gly	0.50	0.11	0.90	0.015	0.046	MLR(a)
Cerebellum	Tau	0.82	0.45	1.19	6.47E-05	3.88E-04	MLR(b)
	Cr + PCr	1.00	0.55	1.46	5.44E-05	3.81E-04	MLR(b)
Hippocampus	Asc	0.30	0.05	0.56	0.022	0.067	MLR(a)
	Glc	0.49	0.07	0.91	0.024	0.067	MLR(a)
	Tau	0.80	0.53	1.07	1.11E-07	5.56E-07	MLR(a)
	Cr + PCr	0.47	0.24	0.69	1.21E-04	4.83E-04	MLR(a)
Striatum	GSH	−0.27	−0.52	−0.01	0.045	0.134	MLR(b)

^a^The age-sex interaction was assessed only for metabolites that had significant male vs. female differences prior to multiple testing correction. ^b^Sex coefficient = mean male minus female metabolite concentrations at a reference age (youngest ages available; 4 weeks for striatum, 8 weeks for hippocampus, and 16 weeks for brainstem and cerebellum) and across methods. ^c^MLR(a) – adjusted for categorical age and age-sex interaction; MLR(b) – adjusted for method, categorical age, and age-sex interaction. Color shading highlights statistically significant results.

## 4. Discussion

### 4.1. Overview

By utilizing ^1^H MRS data collected over a 15-year time frame from different brain regions with consistent animal handling procedures and data quality, we evaluated sex differences in neurochemical concentrations in healthy mice on C57BL/6 and related backgrounds. We report small but significant neurochemical differences between male and female mice across most brain regions. Interestingly, the metabolites that showed sex differences, with higher levels in males, are all linked to energy metabolism: Glc is the primary fuel for the brain (Lund-Andersen, 1979), the Cr and PCr system serves as an energy reservoir (Siesjo, 1978), Tau maintains intracellular osmolarity as water is generated from glucose by the tricarboxylic acid (TCA) cycle (van Gelder, 1989), Glu is a metabolite that may indicate the TCA cycle rate through its exchange with the TCA cycle intermediate α-ketoglutarate (Bednarik et al., 2015) and Asc is an antioxidant that controls reactive oxygen species (ROS) produced by mitochondrial respiration (Rice, 2000). The pattern of sex differences was fairly consistent across different brain regions except for the hypothalamus. Finally, significant interactions between sex and age are reported for taurine and total creatine in regions with the largest sample size available at multiple ages.

### 4.2. Data quality across studies

In this first detailed evaluation of sex differences in neurochemical levels measurable by ^1^H MRS in the mouse brain at high field, we used existing data from 7 different studies (Zacharoff et al., 2012; Tkáč et al., 2016, 2021; Friedrich et al., 2018; Kulhanek et al., 2022; McLoughlin et al., 2023; Zarate et al., 2023). All MRS data utilized were acquired using advanced and highly optimized protocols at 9.4 T, including the localization sequence, voxel-based adjustment of B_0_ homogeneity (B_0_ shimming) and voxel-based calibration of transmitted RF power. The FASTMAP technique in combination with a strong 2^nd^-order shim system (shim coils and drivers) was utilized for B_0_ shimming in all studies, resulting in linewidths in the “excellent” category for mouse models as defined by expert consensus (Juchem et al., 2021). These excellent, adequate, and acceptable linewidth categories are magnetic field-and region-specific because of the well-known differences in microscopic tissue properties that define the best achievable (i.e., intrinsic) linewidths across different brain regions. While the spectral linewidths provided by LCModel (Supplementary Table S1) appear to be underestimated by a few Hertz relative to true linewidths, they nonetheless allow a reliable comparison of spectral quality between regions. The broader linewidths in the brainstem are an indication of higher levels of microscopic magnetic field inhomogeneities over the VOI relative to other regions and vice versa for the striatum and hippocampus.

In interpreting the findings, it is important to consider that the overall detection sensitivity and statistical power were different between brain regions. The spectral signal-to-noise ratio was affected by the VOI size, the distance of the VOI from the RF coil, the best achievable linewidth based on tissue properties of each region, the number of averages and the type of localization sequence (STEAM or LASER). Consequently, some of weakly represented metabolites (Provencher, 2001), such as Ala, Asc, Asp, GSH, Gly, NAAG, and PE were not consistently quantified across brain regions with the CRLB criteria used and therefore were omitted from the final analysis (Figure 2; Supplementary Table S2). In addition, the number of MR spectra available was different between brain regions, e.g., hippocampus data were available from 46 females and 46 males, while hypothalamus data were available from only 5 females and 6 males. Therefore, mean male–female differences that were of comparable magnitude to other VOI were not statistically significant for VOI with small N, e.g., Glu and Tau in the hypothalamus. Similarly, the sample size at specific ages was small for the striatum and cortex, however striatum data were available from two studies that covered 7 different ages and cortex data were available from 4 different ages. Because the direction of the sex differences was consistent across ages, having data at multiple ages increased the statistical power for the striatum and cortex. Still, none of the trends in the cortex survived the Holm-Bonferroni correction. To allow readers evaluate all regional neurochemical differences between sexes, we provide both multiple testing adjusted and unadjusted *p*-values (Figures 2, 3; Supplementary Table S2). The adjusted p-values thereby highlight the most robust findings in this study.

Finally, we used the water signal collected from the same VOI as the metabolites as a quantification reference. The total creatine signal is frequently used as a reference metabolite in ^1^H MRS studies, however the findings regarding sex differences would have been very different if Cr + PCr was used as a reference here because total creatine level was higher in males in four of the six brain regions investigated. This further emphasizes the caution that needs to be taken when interpreting creatine-referenced MRS findings.

### 4.3. Sex differences in neurochemical levels

The sex differences we observed were rather consistent across regions for Asc, Glc, Tau, Glu and Cr + PCr (Figures 2, 3). These data were collected in different studies and from mice with somewhat different genetic backgrounds, indicating that these findings are generalizable. The reproducibility across regions further indicates that the sex differences are not related to specific behaviors that are regionally controlled and divergent between sexes. Of the 321 datasets used, only 16 were collected from young mice (4 weeks of age for cortex and striatum, Table 1). Therefore, these differences were largely observed in adult animals that were matched well for age and environment in each study. That all 5 metabolites were higher in males may raise the question if the differences resulted from different water content in male vs. female mice. Such sex differences in brain water content would have affected all metabolites however, yet significant concentration differences were only observed for a subgroup of metabolites. Mean concentrations of some strongly represented metabolites (low CRLB), such as NAA or myo-Ins were statistically indistinguishable between male and female mice in most studied brain regions. Similarly, the macromolecule content was the same between male and female mice in all brain regions (data not shown). Moreover, a trend for lower Gln in most brain regions further demonstrates that water scaling does not explain the higher concentrations of five metabolites (Asc, Glc, Glu, Tau, Cr + PCr) consistently observed in male mice. Importantly, the higher Tau level in males was also reported in a prior study where water content was explicitly measured postmortem (Duarte et al., 2014). Any age trends are also not explained by changes in brain water content as water content does not change within the age range included here (Duarte et al., 2014). Effects of isoflurane anesthesia need to be considered when interpreting the findings, especially with respect to higher glucose levels in male mice. It is well known that isoflurane anesthesia raises brain lactate (Valette et al., 2007) and blood and brain glucose levels (Kofke et al., 1987), which results in a higher variability in Lac and Glc measurements than other metabolites (Figure 2). The higher Glc levels in males however are consistently observed in all VOI, except for hypothalamus, and therefore unlikely to be a confound of anesthesia. Finally, these studies did not control for the estrous cycle, therefore hormone-regulated changes in metabolite levels in female mice would not have been captured in our analysis. However, the data variability in female mice was identical to that in male mice (Figure 2; Supplementary Table S2), indicating that cycle stage does not modulate MRS-detected neurochemical concentrations. This is consistent with the body of literature showing that most neurobiological measures that display sex differences are not driven by changes in female cyclicity (Bale and Epperson, 2017; Mauvais-Jarvis et al., 2017).

Intriguingly, all five metabolites that are consistently higher in males are directly or indirectly associated with cerebral energy metabolism, therefore the observed differences may reflect overall metabolic differences between males and females. Namely, glucose is the primary fuel for the brain (Lund-Andersen, 1979) and creatine and phosphocreatine are essential in maintaining ATP homeostasis (Siesjo, 1978). Ascorbate, a key intracellular antioxidant, scavenges ROS generated by mitochondrial oxidative metabolism (Rice et al., 2002). The primary excitatory neurotransmitter glutamate is intimately involved in energy metabolism (Öz et al., 2012) and its concentration increases dynamically upon brain activation as an indicator of increased oxidative metabolism (Mangia et al., 2007; Bednarik et al., 2015). While taurine is a relatively inert metabolite, it displays a positive correlation with cerebral metabolic rate for glucose (CMR_glc_) across species, likely due to its osmoregulatory role to compensate for glucose derived water production by oxidative phosphorylation in mitochondria (van Gelder, 1989). The higher levels of these metabolites, particularly glutamate, ascorbate, and taurine, in male mice would therefore indicate higher oxidative energy metabolism in males. However, prior work in humans (Andreason et al., 1994; Shin et al., 2021) and mice (Gaignard et al., 2018) has shown that CMR_glc_ and mitochondrial metabolism are overall higher in females than males. A higher flux of glucose through glycolysis and TCA cycle would explain the lower Glc levels in female mice if transport through the blood brain barrier is comparable. In addition, higher ATP/PCr levels were reported in women than men, which was interpreted as higher ATP production and utilization in women (Jett et al., 2022). A lower PCr level in the ATP/PCr ratio is consistent with our lower Cr + PCr finding in females, although our sum measure does not distinguish between Cr and PCr. A higher total creatine level, if it is due to higher PCr, may indicate lower energy utilization in males, consistent with lower cerebral metabolic rates relative to females (Gaignard et al., 2018).

An alternative explanation for higher glutamate, ascorbate and taurine in males may involve considerations of cellular, and particularly neuronal, density (number of neurons per mm^3^). A higher cellular density in males was recently reported in many brain regions in a large mouse population (Elkind et al., 2023). A higher average neuronal density in the cerebral cortex of men than women was also reported by histology (Rabinowicz et al., 2002). Also, diffusion MRI showed a higher intracellular volume fraction in males vs. females, which may result from a higher neuronal density (Kodiweera et al., 2016). Therefore, the higher Tau levels may indicate a higher osmoregulatory capacity in male mice, necessary to support a higher cell density. Ascorbate was higher in cortex, hippocampus and cerebellum in male mice (Figure 3), which is in agreement with previously reported results in rat brain tissue (Kume-Kick and Rice, 1998). Asc is primarily neuronal and correlates with neuronal density across species (Rice and Russo-Menna, 1998; Rice et al., 2002). Finally, the trend for higher glutamate in all brain regions, together with a more modest trend for lower glutamine in the striatum, hippocampus and cortex are consistent with a higher neuron-to-glia ratio in male mice because glutamate is primarily localized in neurons and glutamine primarily in glia (Öz et al., 2012). Interestingly, the neuron-to-glia ratio is positively correlated with neuronal density across brain structures and species (Herculano-Houzel, 2014). Taken together, this pattern (higher Tau, Asc, Glu and lower Gln) may be due to a higher neuronal density in male mice. In this context, the lack of a sex difference for NAA, except for the brainstem, provides further support for NAA being an indicator of neuronal function, rather than cell count. Namely, NAA is localized exclusively in neurons in the adult brain (Öz et al., 2014). However, a larger number of neurons in an MRS voxel does not necessarily imply higher NAA concentration. For example, NAA declines in the early phases of neurodegeneration, during neuronal dysfunction, but levels out during later periods of cell loss (Öz et al., 2010b), indicating that is a marker of neuronal function rather than cell loss.

Similar to ascorbate, taurine also has antioxidant and neuroprotective properties (Wu and Prentice, 2010), which may result from its osmoregulatory role, such as by reducing the glutamate-induced increase of intracellular calcium (Schaffer et al., 2000). A taurine deficit is one of the earliest neurochemical changes in some neurodegenerative disease models (Öz et al., 2010a; Rafiee et al., 2022), even before overt pathology (Emir et al., 2013). In addition, taurine supplementation has shown beneficial effects in animal models of neurodegeneration (Jakaria et al., 2019). Therefore, it is tempting to speculate that the lower Tau and Asc levels in females may be factors that increase their risk for neurodegeneration (Alzheimer’s Association, 2021). Namely, these data indicate that females have lower antioxidant and osmoregulatory capacity despite higher reported oxidative metabolic rates, which would generate higher levels of reactive oxygen species and water.

The pattern for sex differences in the hypothalamus remains to be confirmed at other ages. This neuroendocrine structure consists of a set of different nuclei with different functions and metabolic properties, potentially blurring sex differences in metabolite levels. The higher levels of choline-containing compounds in females is likely linked to the central role of the hypothalamus in sensing and integrating glucose and lipid metabolism related signals, including hormonal, as well as central and peripheral neuronal inputs (Jordan et al., 2010).

From the translational perspective, these data motivate similar studies in human subjects. One notable difference between the human and mouse brain is the much lower taurine levels in the human brain (Öz et al., 2013). Due to the similarity of the glucose and taurine spectral patterns and resulting high cross-correlations, the sum of glucose and taurine is commonly reported in the human brain. Therefore, potentially higher levels of taurine and glucose in male human subjects should be detectable in the sum of Glc and Tau. Total creatine, ascorbate and glutamate are also reliably quantified in the human brain (Emir et al., 2012; Terpstra et al., 2016) and small sex differences would be detectable with existing high-quality data.

### 4.4. Age-sex interactions in neurochemical levels

Most of the data utilized in this retrospective analysis were obtained after puberty and at peak estrogen levels during the lifetime of female mice, between 2 and 10 months (Finch et al., 1984). The 4-week striatum and cortex data that were available from a limited number of animals were insufficient to draw definitive conclusions about the presence or absence of sex differences at 1 month. To provide some insight into the question whether the neurochemical differences are present from birth or ensue after puberty, we investigated hippocampus data available from P14 (2-week-old) mice collected in another study (Wallin et al., 2015) and observed *no* differences in any metabolite levels between males and females (data not shown). These data were not included in our analysis because the neurochemical profile in the developing mouse brain is very different from the adult brain. Still, this preliminary analysis indicates that the sex differences in the MRS-detectable neurochemicals develop after puberty due to hormonal changes. Consistently, the previously reported sex differences in Asc levels in the rat brain were only present after puberty (Kume-Kick and Rice, 1998). Future prospective studies are needed to confirm that these sex differences are a result of hormonal changes and not present from birth.

### 4.5. Limitations

The main limitation of this study was that the data were not prospectively collected, with consistent sample sizes and ages across VOIs. However, by demonstrating sex differences in neurochemical profiles of healthy mice, this retrospective analysis of a convenience sample provides the impetus for prospectively designed longitudinal studies. Future work should include *in vivo* measurements of regional metabolic fluxes to test the hypotheses generated by the current measurements of steady state metabolite concentrations. In addition, utilizing ^31^P MRS together with ^1^H MRS would allow direct assessments of high energy phosphates such as ATP, ADP and PCr (Zhu et al., 2009).

The age-sex interaction data should be considered preliminary as we had data available from limited and varying age ranges for different brain regions. Therefore, the age trajectories for sex differences should be defined in future studies, especially to understand the underlying mechanisms of the differences.

Guidelines have been outlined for experimental design and interpretation in preclinical studies addressing the mechanisms of sex differences, e.g., by utilizing ovariectomized female and orchiectomized male rodents (McCarthy et al., 2012; Bale and Epperson, 2017; Mauvais-Jarvis et al., 2017). Following these guidelines will provide important insights on sex differences in neurochemistry and metabolism in the future.

## 5. Conclusion

Sex differences in regional neurochemical levels are small but significant and age-dependent, with consistent male–female differences across most brain regions. Hypothalamus, a central hub in the endocrine system, is an exception from the otherwise consistent pattern of sex differences in neurochemical levels. These findings underline the importance of sex-matching control and disease cohorts; otherwise, cohort differences may be observed that stem from sex differences and are not relevant to the disease. Furthermore, based on these data, it is critical to include both sexes when designing experiments, especially in studies addressing metabolism and in preclinical trials that target metabolic deficits, so that findings are relevant to both males and females and therapeutics that may be efficacious in one sex and potentially harmful in the other can be identified at the preclinical stage.

## Data availability statement

The original contributions presented in the study are included in the article/Supplementary materials, further inquiries can be directed to the corresponding author.

## Ethics statement

All experimental protocols included in this study were approved by the Institutional Animal Care and Use Committee at the University of Minnesota. The study was conducted in accordance with the local legislation and institutional requirements.

## Author contributions

IT: Conceptualization, Data curation, Funding acquisition, Investigation, Writing – original draft, Writing – review & editing, Formal Analysis, Methodology. TX: Formal analysis, Writing – original draft, Writing – review & editing. NS: Formal Analysis, Writing – original draft, Writing – review & editing. SL: Writing – review & editing, Investigation. JD: Investigation, Writing – review & editing, Funding acquisition. RG-P: Investigation, Writing – review & editing, Funding acquisition. HM: Investigation, Writing – review & editing, Funding acquisition. HO: Investigation, Writing – review & editing, Funding acquisition. LE: Investigation, Writing – review & editing, Conceptualization, Supervision. GÖ: Conceptualization, Investigation, Supervision, Writing – review & editing, Data curation, Funding acquisition, Writing – original draft.
