# Supplementary material for: Regional sex differences in neurochemical profiles of healthy mice measured by magnetic resonance spectroscopy at 9.4 tesla
Source: Front Neurosci. 2023 Oct 25;17:1278828. doi: 10.3389/fnins.2023.1278828 (PMC10634209; doi:10.3389/fnins.2023.1278828)
Supplement: Supplementary file 1 [file Table_1.DOCX]

Supplementary Material

**Supplementary Table S1.** Metabolite spectral linewidths (FWHM – full width half maximum) estimated by LCModel for ^1^H MR spectra acquired from different brain regions of male and female WT mice.

|  | **FWHM - Female** | | **FWHM - Male** | |  |
| --- | --- | --- | --- | --- | --- |
| **Brain region** | **N**^a^ | **Mean ± SD (Hz)** | **N**^a^ | **Mean ± SD (Hz)** | **p-value**^b^ |
| Brainstem | 25 | 11.3 ± 2.9 | 34 | 10.9 ± 2.0 | 0.579 |
| Cerebellum | 32 | 9.5 ± 2.4 | 40 | 9.3 ± 1.6 | 0.652 |
| Cortex | 14 | 7.2 ± 1.6 | 16 | 7.4 ± 2.2 | 0.751 |
| Hippocampus | 46 | 6.4 ± 0.7 | 46 | 6.4 ± 0.9 | 1.000 |
| Hypothalamus | 5 | 9.2 ± 3.3 | 6 | 8.5 ± 2.2 | 0.683 |
| Striatum | 33 | 6.2 ± 1.5 | 24 | 5.7 ± 1.3 | 0.202 |

^a^ N – number of spectra in each group

^b^ P-values obtained from unpaired, two-tailed t-tests.

**Supplementary Table S2.** Predicted regional neurochemical concentrations (µmol/g) and differences between male and female WT mice at an example age (brainstem: 18 weeks; cerebellum: 16 weeks; cortex: 12 weeks; hippocampus: 12 weeks; hypothalamus: 16 weeks; striatum: 12 weeks) across methods.

| Metabolite | Male  Mean (SE)^a^ | Female  Mean (SE)^a^ | Mean  Difference (CI)^b^ | p-value^c^ | Adjusted  p-value^c^ |
| --- | --- | --- | --- | --- | --- |
| Brainstem |  |  |  |  |  |
| Asp | 3.32 (0.32) | 3.24 (0.36) | 0.09 (-0.51, 0.69) | 0.773 | 1 |
| GABA | 2.13 (0.21) | 1.93 (0.23) | 0.21 (-0.14, 0.55) | 0.246 | 1 |
| Glc | 2.98 (0.33) | 1.96 (0.38) | 1.02 (0.38, 1.66) | **0.0035** | **0.045** |
| Gln | 2.28 (0.23) | 2.12 (0.25) | 0.16 (-0.21, 0.53) | 0.405 | 1 |
| Glu | 5.01 (0.21) | 4.81 (0.23) | 0.20 (-0.14, 0.54) | 0.252 | 1 |
| Gly | 3.28 (0.13) | 3.02 (0.14) | 0.26 (0.05, 0.47) | **0.0187** | 0.224 |
| myo-Ins | 5.53 (0.23) | 5.45 (0.25) | 0.08 (-0.30, 0.45) | 0.695 | 1 |
| Lac | 4.66 (0.35) | 4.34 (0.38) | 0.32 (-0.26, 0.90) | 0.283 | 1 |
| NAA | 7.30 (0.25) | 6.85 (0.27) | 0.45 (0.05, 0.86) | **0.0311** | 0.342 |
| NAAG | 0.63 (0.10) | 0.69 (0.11) | -0.07 (-0.24, 0.10) | 0.454 | 1 |
| Tau | 3.02 (0.22) | 2.66 (0.24) | 0.36 (0.00, 0.72) | 0.052 | 0.524 |
| GPC+PC | 1.13 (0.07) | 1.03 (0.08) | 0.09 (-0.02, 0.21) | 0.117 | 1 |
| Cr+PCr | 6.61 (0.22) | 6.36 (0.24) | 0.25 (-0.12, 0.61) | 0.189 | 1 |

| Metabolite | Male  Mean (SE)^a^ | Female  Mean (SE)^a^ | Mean  Difference (CI)^b^ | p-value^c^ | Adjusted  p-value^c^ |
| --- | --- | --- | --- | --- | --- |
| Cerebellum |  |  |  |  |  |
| Asc | 2.28 (0.15) | 1.85 (0.15) | 0.42 (0.22, 0.63) | **1.27E-04** | **1.27E-03** |
| GABA | 2.32 (0.12) | 2.29 (0.12) | 0.03 (-0.13, 0.20) | 0.705 | 1 |
| Glc | 3.69 (0.37) | 2.60 (0.37) | 1.09 (0.58, 1.60) | **8.84E-05** | **9.73E-04** |
| Gln | 3.58 (0.12) | 3.65 (0.12) | -0.07 (-0.24, 0.10) | 0.417 | 1 |
| Glu | 8.86 (0.15) | 8.56 (0.15) | 0.30 (0.09, 0.51) | **7.48E-03** | 0.060 |
| GSH | 1.31 (0.08) | 1.22 (0.08) | 0.08 (-0.03, 0.20) | 0.142 | 0.566 |
| myo-Ins | 6.85 (0.15) | 6.57 (0.14) | 0.29 (0.09, 0.49) | **6.67E-03** | 0.060 |
| Lac | 2.88 (0.21) | 2.60 (0.20) | 0.28 (-0.01, 0.56) | 0.062 | 0.369 |
| NAA | 8.08 (0.11) | 8.06 (0.11) | 0.02 (-0.13, 0.18) | 0.771 | 1 |
| NAAG | 0.75 (0.05) | 0.81 (0.05) | -0.07 (-0.14, 0.01) | 0.075 | 0.375 |
| Tau | 8.32 (0.14) | 7.39 (0.14) | 0.93 (0.73, 1.12) | **8.10E-14** | **1.05E-12** |
| GPC+PC | 1.77 (0.05) | 1.67 (0.05) | 0.10 (0.03, 0.17) | **8.87E-03** | 0.062 |
| Cr+PCr | 12.40 (0.17) | 11.51 (0.16) | 0.89 (0.66, 1.12) | **1.17E-10** | **1.40E-09** |

| Metabolite | Male  Mean (SE)^a^ | Female  Mean (SE)^a^ | Mean  Difference (CI)^b^ | p-value^c^ | Adjusted  p-value^c^ |
| --- | --- | --- | --- | --- | --- |
| Cortex |  |  |  |  |  |
| Ala | 1.07 (0.11) | 1.14 (0.12) | -0.07 (-0.28, 0.15) | 0.534 | 1 |
| Asc | 2.51 (0.18) | 2.08 (0.17) | 0.43 (0.12, 0.74) | **0.013** | 0.193 |
| GABA | 1.94 (0.15) | 1.86 (0.15) | 0.08 (-0.20, 0.35) | 0.594 | 1 |
| Glc | 4.44 (0.29) | 3.77 (0.29) | 0.67 (0.13, 1.20) | **0.022** | 0.267 |
| Gln | 2.61 (0.20) | 3.01 (0.20) | -0.39 (-0.76, -0.03) | **0.042** | 0.384 |
| Glu | 10.78 (0.30) | 10.17 (0.30) | 0.61 (0.06, 1.16) | **0.038** | 0.384 |
| GSH | 1.03 (0.09) | 1.06 (0.08) | -0.04 (-0.18, 0.12) | 0.656 | 1 |
| myo-Ins | 5.35 (0.18) | 5.45 (0.18) | -0.10 (-0.42, 0.22) | 0.546 | 1 |
| Lac | 1.75 (0.47) | 2.09 (0.47) | -0.34 (-1.20, 0.52) | 0.445 | 1 |
| NAA | 8.75 (0.21) | 8.69 (0.21) | 0.07 (-0.32, 0.45) | 0.743 | 1 |
| NAAG | 1.04 (0.13) | 1.10 (0.13) | -0.06 (-0.30, 0.18) | 0.616 | 1 |
| PE | 1.94 (0.19) | 2.21 (0.19) | -0.27 (-0.62, 0.07) | 0.133 | 1 |
| Tau | 10.22 (0.25) | 9.62 (0.25) | 0.60 (0.16, 1.05) | **0.014** | 0.193 |
| GPC+PC | 1.31 (0.05) | 1.20 (0.05) | 0.11 (0.03, 0.20) | **0.013** | 0.193 |
| Cr+PCr | 8.44 (0.13) | 8.14 (0.13) | 0.29 (0.05, 0.53) | **0.025** | 0.279 |

| Metabolite | Male  Mean (SE)^a^ | Female  Mean (SE)^a^ | Mean  Difference (CI)^b^ | p-value^c^ | Adjusted  p-value^c^ |
| --- | --- | --- | --- | --- | --- |
| Hippocampus |  |  |  |  |  |
| Asc | 2.72 (0.08) | 2.51 (0.08) | 0.20 (0.03, 0.37) | **0.023** | 0.230 |
| GABA | 1.68 (0.06) | 1.63 (0.06) | 0.05 (-0.07, 0.17) | 0.383 | 1 |
| Glc | 3.27 (0.13) | 2.60 (0.13) | 0.67 (0.40, 0.95) | **6.53E-06** | **7.84E-05** |
| Gln | 3.45 (0.08) | 3.62 (0.08) | -0.17 (-0.35, 0.01) | 0.070 | 0.626 |
| Glu | 9.90 (0.09) | 9.66 (0.09) | 0.25 (0.06, 0.44) | **0.013** | 0.148 |
| GSH | 1.04 (0.04) | 1.01 (0.04) | 0.03 (-0.05, 0.11) | 0.522 | 1 |
| myo-Ins | 6.50 (0.08) | 6.55 (0.08) | -0.05 (-0.22, 0.12) | 0.575 | 1 |
| Lac | 2.56 (0.11) | 2.63 (0.11) | -0.07 (-0.31, 0.17) | 0.567 | 1 |
| NAA | 8.40 (0.06) | 8.32 (0.06) | 0.08 (-0.04, 0.21) | 0.202 | 1 |
| NAAG | 0.72 (0.03) | 0.71 (0.03) | 0.01 (-0.06, 0.08) | 0.753 | 1 |
| PE | 3.18 (0.08) | 3.25 (0.08) | -0.07 (-0.24, 0.10) | 0.414 | 1 |
| Tau | 12.84 (0.08) | 11.81 (0.08) | 1.03 (0.85, 1.22) | **2.80E-18** | **3.92E-17** |
| GPC+PC | 1.02 (0.02) | 1.02 (0.02) | 0.00 (-0.05, 0.05) | 0.865 | 1 |
| Cr+PCr | 10.88 (0.07) | 10.40 (0.07) | 0.48 (0.33, 0.63) | **8.77E-09** | **1.14E-07** |

| Metabolite | Male  Mean (SE)^a^ | Female  Mean (SE)^a^ | Mean  Difference (CI)^b^ | p-value^c^ | Adjusted  p-value^c^ |
| --- | --- | --- | --- | --- | --- |
| Hypothalamus |  |  |  |  |  |
| Asc | 2.30 (0.26) | 2.30 (0.29) | -0.01 (-0.86, 0.84) | 0.986 | 1 |
| Asp | 2.15 (0.20) | 2.21 (0.22) | -0.06 (-0.68, 0.55) | 0.843 | 1 |
| GABA | 4.84 (0.17) | 4.77 (0.18) | 0.07 (-0.43, 0.57) | 0.794 | 1 |
| Glc | 2.34 (0.37) | 2.43 (0.41) | -0.09 (-1.22, 1.05) | 0.884 | 1 |
| Gln | 4.25 (0.25) | 3.75 (0.27) | 0.49 (-0.27, 1.26) | 0.241 | 1 |
| Glu | 6.92 (0.19) | 6.49 (0.20) | 0.43 (-0.11, 0.98) | 0.145 | 1 |
| GSH | 0.78 (0.12) | 0.95 (0.13) | -0.18 (-0.54, 0.19) | 0.373 | 1 |
| myo-Ins | 9.46 (0.24) | 9.55 (0.26) | -0.10 (-0.87, 0.68) | 0.815 | 1 |
| Lac | 4.59 (0.27) | 4.64 (0.29) | -0.05 (-0.82, 0.71) | 0.893 | 1 |
| NAA | 6.05 (0.16) | 6.13 (0.17) | -0.08 (-0.57, 0.41) | 0.761 | 1 |
| NAAG | 0.90 (0.10) | 1.01 (0.12) | -0.11 (-0.44, 0.22) | 0.548 | 1 |
| PE | 2.33 (0.30) | 1.85 (0.33) | 0.48 (-0.43, 1.39) | 0.322 | 1 |
| Tau | 5.07 (0.24) | 4.35 (0.26) | 0.72 (0.03, 1.41) | 0.066 | 0.928 |
| GPC+PC | 2.24 (0.05) | 2.44 (0.05) | -0.20 (-0.35, -0.05) | **0.037** | 0.548 |
| Cr+PCr | 6.65 (0.17) | 6.64 (0.19) | 0.01 (-0.53, 0.55) | 0.967 | 1 |

| Metabolite | Male  Mean (SE)^a^ | Female  Mean (SE)^a^ | Mean  Difference (CI)^b^ | p-value^c^ | Adjusted  p-value^c^ |
| --- | --- | --- | --- | --- | --- |
| Striatum |  |  |  |  |  |
| Ala | 1.53 (0.12) | 1.51 (0.12) | 0.02 (-0.16, 0.19) | 0.856 | 1 |
| GABA | 2.38 (0.13) | 2.46 (0.13) | -0.08 (-0.27, 0.11) | 0.400 | 1 |
| Glc | 4.68 (0.31) | 4.31 (0.31) | 0.37 (-0.09, 0.83) | 0.124 | 1 |
| Gln | 3.61 (0.13) | 3.73 (0.13) | -0.13 (-0.32, 0.06) | 0.194 | 1 |
| Glu | 8.78 (0.23) | 8.37 (0.23) | 0.41 (0.08, 0.73) | **0.019** | 0.223 |
| GSH | 1.34 (0.07) | 1.45 (0.07) | -0.12 (-0.22, -0.01) | **0.037** | 0.404 |
| myo-Ins | 5.37 (0.18) | 5.30 (0.18) | 0.07 (-0.19, 0.33) | 0.587 | 1 |
| Lac | 5.46 (0.61) | 5.54 (0.61) | -0.08 (-0.97, 0.82) | 0.870 | 1 |
| NAA+NAAG | 7.74 (0.16) | 7.76 (0.16) | -0.02 (-0.25, 0.21) | 0.881 | 1 |
| PE | 3.69 (0.16) | 3.85 (0.16) | -0.17 (-0.40, 0.07) | 0.174 | 1 |
| Tau | 13.74 (0.28) | 12.85 (0.28) | 0.89 (0.48, 1.30) | **9.79E-05** | **1.27E-03** |
| GPC+PC | 1.84 (0.06) | 1.75 (0.06) | 0.09 (0.00, 0.18) | 0.064 | 0.635 |
| Cr+PCr | 8.75 (0.14) | 8.71 (0.14) | 0.04 (-0.17, 0.25) | 0.706 | 1 |

^a^ Concentrations predicted by the statistical models (hippocampus, cortex and brainstem values adjusted for categorical age; striatum and cerebellum values adjusted for MRS method and categorical age; hypothalamus values unadjusted) are shown together with their standard errors (SE) in parentheses.

^b^ The mean differences and 95% confidence intervals (CI) represent average concentration difference (male minus female) at any age.

^c^ The p-values listed are associated with the mean difference in concentration and are Holm-Bonferroni corrected for multiple testing across metabolites within region.
